# Population Dynamics of Fall Armyworm (Lepidoptera: Noctuidae) in Maize Fields in Uganda

**DOI:** 10.3390/insects15050301

**Published:** 2024-04-23

**Authors:** Angella Lowra Ajam, Jeninah Karungi, Geofrey Ogwal, Stella Aropet Adumo, Pamela Paparu, Michael Hilary Otim

**Affiliations:** 1 Department of Agricultural Production, College of Agricultural and Environmental Sciences, Makerere University, Kampala P.O. Box 7062, Uganda; jtumutegyereize@gmail.com; 2 National Agriculture Research Organisation, National Crops Resources Research Institute–Namulonge, Kampala P.O. Box 7084, Uganda; pamela.paparu@gmail.com; 3 Alliance of Bioversity International and CIAT, Kampala P.O. Box 24384, Uganda; ogwalgeff1996@gmail.com; 4 National Agriculture Research Organization, National Agricultural Research Laboratories, Kawanda, Kampala P.O. Box 7065, Uganda; stellaadumo@yahoo.com

**Keywords:** cropping system, pesticide use, rainfall, temperature, tillage system

## Abstract

**Simple Summary:**

Fall armyworm (FAW) was first detected in Uganda in 2016 and has spread to all the maize-growing districts. Different methods have been deployed to control this pest. However, there is a limited understanding of the role of the environment and farmers’ practices on the abundance of and damage by *S. frugiperda* in Uganda. In this study, we aimed to assess the abundance of *S. frugiperda* and leaf damage levels in three different districts. We explored the association between crop management practices, crop stage, and weather parameters on abundance of and damage by *S. frugiperda* in smallholder farmers’ maize fields using a longitudinal monitoring survey in 69 farmers’ fields of Kole, Kiryandongo, and Nakaseke for three seasons. The numbers of egg masses and adults were generally low. The highest numbers of adults were trapped in Kiryandongo, followed by Nakaseke, and the lowest numbers were trapped in Kole. Leaf damage and incidence of damaged plants differed in the different seasons and districts. Conservation tillage, reduced weeding frequency, increase in rainfall and high maximum temperature reduced *S. frugiperda* leaf damage. There was no relationship between pesticide use frequency and cropping system with *S. frugiperda* leaf damage. However, the influence of fertilizer use on leaf damage was contradictory across seasons and districts. Timely and vigilant scouting, proper timing of control measures, minimum tillage practices, and crop diversity should be included in integrated management for *S. frugiperda*.

**Abstract:**

*Spodoptera frugiperda* (Lepidoptera: Noctuidae), commonly known as fall armyworm, was first detected in Uganda in 2016 and has spread to all the maize-growing districts. Different methods have been deployed to control this pest. However, there is a limited understanding of the role of the environment and farmers’ practices on the abundance of and damage by *S. frugiperda* in Uganda. This study, therefore, assessed the abundance of *S. frugiperda* and leaf damage levels in three different districts and explored the association between agronomic practices, crop phenology, and weather parameters on *S. frugiperda* damage and abundance in smallholder farmers’ maize fields using a longitudinal monitoring survey in 69 farmers’ fields of Kole, Kiryandongo, and Nakaseke for three seasons. The numbers of egg masses and adults were generally low. The highest numbers of adults were trapped in Kiryandongo, followed by Nakaseke, and the lowest numbers were trapped in Kole. Leaf damage and incidence of damaged plants differed significantly between districts and seasons. Leaf damage and abundance of larvae varied significantly in the districts and at different growth stages. Conservation tillage, reduced weeding frequency, increased rainfall and high maximum temperatures were associated with reduced *S. frugiperda* damage. No significant relationship was observed between pesticide or cropping systems with *S. frugiperda* leaf damage. However, the influence of fertilizer use on leaf damage was contradictory across seasons and districts. Timely and vigilant scouting, proper timing of control measures, and minimum tillage practices should be included in an IPM strategy for *S. frugiperda***.**

## 1. Introduction

Maize is one of the three most important cereals for food security globally and is particularly important in the diets of the poor in Africa and Latin America [1]. Maize is a key income earner for farmers and a source of foreign exchange for the government of Uganda. Over the last decade, Uganda has earned about US$75 m annually from maize exports [2]. Productivity growth has not been in line with the ever-increasing population and the demand for maize for food, feed, and industrial materials due to biotic and abiotic pressures. The pressures include drought, heat, poor soil fertility, and waterlogging/excess moisture [3], often coupled with diseases [4] and insect pests. Arthropod pests are among the key factors contributing to low yields of maize. These include the maize stalk borers *Busseola fusca* (Fuller, 1901, Lepidoptera: Noctuidae) and the sported stemborer *Chilo partellus* (Swinhoe, 1885, Lepidoptera: Crambidae), cutworms and weevils [5]. *Spodoptera frugiperda* (J.E. Smith, Lepidoptera: Noctuidae), commonly referred to as fall armyworm (FAW), is now a significant insect pest that was first reported in Africa in early 2016 [6].

*Spodoptera frugiperda* has a high potential for rapid spread and poses a real threat to the food security and livelihoods of millions of smallholder maize farmers in Africa. A study by Abrahams et al. [7] showed that the pest could cause annual maize losses of 80–200 million tonnes in 12 maize-producing countries in sub-Saharan Africa without effective control. In Uganda, it can potentially cause a loss in yield of up to 52% [8]. *Spodoptera frugiperda* attacks all crop stages and causes severe leaf damage and direct damage to maize ears. The larvae defoliate and can kill young plants or the young whorl of plants, resulting in a dead heart [9].

Host plant resistance, cultural, biological, botanical, chemical, and biotechnological approaches have been used to manage *S. frugiperda* [10]. The agronomic and cultural approaches include early planting, adequate nutrient supply through mineral fertilizer, intercropping, frequent weeding, proper tillage, and pheromone traps. Farmers also use innovations such as ash, chilli, sand, sugar solutions, and fish soup [11,12]. In response to the enormous threat of crop yield losses from the invasive *S. frugiperda*, the government of Uganda has promoted the use of synthetic insecticides, e.g., Striker (Lamba Cyhalothrin and Thiamethoxam), Roket (Profenofos and Cypermethrin) for its control on maize. However, the results of the effectiveness of the pesticides are variable and inconclusive. Moreover, chemical insecticides present a hazard to users, the environment, and consumers. Also, the farmers’ use of insecticides has not been guided by proper ecological considerations related to population dynamics, particularly knowledge of factors affecting population density, damage, and abundance. Many factors, including farmers’ agricultural practices such as pesticide use, fertilizer use, weeding frequency, cropping and tillage system [13], and environmental factors such as rainfall and temperature, are key to understanding the population dynamics of *S. frugiperda* [14]. There has not been any systematic season-long follow-up of the abundance of *S. frugiperda* in Uganda. Therefore, this study aimed to assess population dynamics and damage by *S. frugiperda* as influenced by farmers’ practices and weather conditions in three districts of Uganda.

## 2. Materials and Methods

The study was conducted in Nakaseke, Kole, and Kiryandongo districts, which lie in three different agro-ecologies of Uganda. Nakaseke, Kole, and Kiryandongo lie in western savannah grasslands, northeastern savannah grasslands, and northwestern savannah grasslands, respectively (Table 1). The districts are one of the major producers of maize in the respective agro-ecologies [15]. Additionally, unpublished results of a survey carried out in 2020 showed these districts as having high *S. frugiperda* damage [16].

### 2.1. Study Design

**Figure 1:**
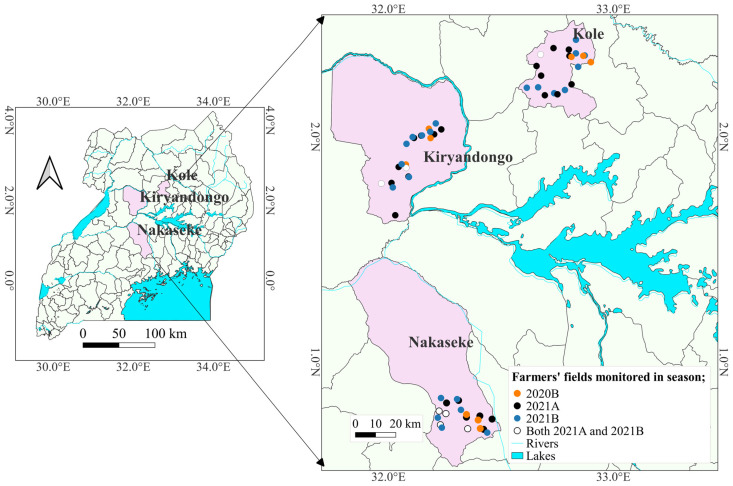
A map of Uganda showing the districts (left panel) and farmers’ fields where the population dynamics of *Spodoptera frugiperda* were studied. Each year’s first and second rainy seasons are distinguished by the letters A and B, respectively.

A longitudinal monitoring survey was conducted in Kole, Kiryandongo, and Nakaseke districts (Figure 1) for three seasons: 2020B (September to December), 2021A (March to July), and 2021B (September to December). Each year’s first and second rainy seasons are distinguished by the letters A and B, respectively. In each of the districts, ten maize fields (2021A and 2021B) and three fields in 2020B were selected purposively based on the planting date and farmers’ consent to interviews and access to the field. A total of 69 fields were monitored. The difference in the number of fields in the different years was because of fund availability. The fields measured at least one acre and were separated by at least 5 km. The GPS location of each field was recorded using GPS Test App version 1.6.3. All fields were managed entirely by the farmers.

### 2.2. Abundance and Damage of Spodoptera frugiperda Life Stages

Data collection began approximately three weeks after planting (WAP) and continued every two weeks till harvest. On every visit, the phenological stage of the maize plants was obtained using the ‘leaf collar method’ [17] and recorded. To determine the abundance of *S. frugiperda* in the field, each field was divided into four quadrants measuring approximately 0.125 acres, and 15 maize plants were sampled randomly in each, making a total of 60 plants per field. Plants within five meters of the edge were not sampled, to avoid edge effects. Each sampled plant was examined for the presence of *S. frugiperda* eggs, larvae, and pupae and scored for *S. frugiperda* leaf damage. The number of life stages on each plant was counted and recorded. Leaf damage was scored on a scale of 0–9 according to [18], where 0 = No visual leaf injury and 9 = Whorl and furl leave almost destroyed.

Adult populations were also monitored using the pheromone traps deployed in universal bucket traps set up in each farmer’s field a month after planting. The P061 pheromone containing Z11-hexadecenyl acetate and Z9-tetradecenyl acetate (4.35 g a.i/kg), manufactured by Chemtica Internacional S.A., was used. The traps were hung upright on a long pole at 1.2 m off the ground. The pheromone lure was placed on the top section of the bucket trap and replaced every four weeks based on the manufacturer’s recommendation. The trapped adults were counted and recorded every two weeks until harvest.

### 2.3. Farmers’ Practices

We interviewed owners of the selected farms to obtain information on fertilizer use (yes or no), pesticide use frequency, tillage system, and weeding frequency. Conservation tillage is defined as zero tillage where there was no tillage and herbicides were used to kill weeds before planting maize. Conventional tillage is when the land is opened and fine-tilled using an ox plough, hoe, or tractor. The cropping system was observed and recorded. The cropping system was defined as sole or intercropped.

### 2.4. Environmental Parameters

Mean daily minimum and maximum temperature and total rainfall were sourced from the Uganda Meteorological Authority.

### 2.5. Data Analysis

The means of leaf damage, damage incidence (proportion of damaged plants), and the mean numbers of egg masses, larvae, and trapped adults were tested for normality using the Shapiro–Wilk test and Levene test for equal variance using the ‘car’ package of R Studio using R version 4.2.1 [19]. Data on the number of egg masses, larvae and adults trapped were transformed by powers 0.175, 0.25 and 0.3, respectively, using Tukey’s Ladder of Powers procedure. We performed a general ANOVA on number of egg masses and larvae, leaf damage, and leaf damage incidence, where the factors included district, growth stage, season nested within districts and field nested within a season.

Further, downstream analysis was carried out for each district to determine seasonal differences in the mean leaf damage, damage incidence, mean number of egg masses, larvae, and trapped adults, using general ANOVA with growth stage and fields nested within season as factors. Mean separations were carried out using Fisher’s LSD.

Leaf damage incidence (percentage damage) for *S. frugiperda* was calculated as a proportion (%) of the total plants sampled that had leaf damage symptoms.

Graphs on the mean number of larvae per 15 plants and mean leaf damage over the different growth stages were plotted; however, mean number of egg masses was not calculated because of the very low numbers.

The categorical data on the different management practices were coded using the dummy coding method. Their dummy variables were used to perform the multiple regression analysis in R studio using the “lm”() function to establish the relationship between management practices and weather factors (rainfall and maximum temperature) with the mean number of larvae per 15 plants and mean leaf damage in the different seasons and districts. The management practices, except pesticide application frequency and weeding, were coded. The following were baseline variables for each management practice: fertilizer use; no—0, tillage system; conservation—0, cropping system; sole—0. These baseline variables were used because they occurred most often. Relationships between management practices plus weather factors and the number of egg masses and larvae were not studied because they were too low to establish this relationship.

## 3. Results

### 3.1. Abundance and Damage of Spodoptera frugiperda Life Stages in the Study Districts

The number of egg masses was low on all sampling dates in the three districts and did not differ significantly between the districts (F_2,167_ = 1.14, *p* = 0.323). The number of egg masses ranged from 0.01 per 15 plants in Kiryandongo to 0.03 masses per 15 plants in Kole. There were, however, significant differences in the numbers of egg masses between seasons within districts (F_6,167_ = 2.49, *p* = 0.02). The number of egg masses was only significantly different between the seasons in Nakaseke (F_6,49_ = 4.31, *p* = 0.019), where the highest abundance was recorded in 2021A (Table 2).

The abundance of larvae was significantly different between the three districts (F_2,167_ = 6.34, *p* = 0.002) and was highest in Kiryandongo (0.68 ± 0.19 larvae per 15 plants), but similar in Kole (0.25 ± 0.04 larvae per 15 plants) and Nakaseke (0.36 ± 0.09 larvae per 15 plants). Similarly, there were significant differences in the abundance of larvae between seasons in a district (F_2,167_ = 9.75, *p* < 0.001) (Table 2). The highest larval abundance was recorded in 2020B and 2021A in Kiryandongo and Nakaseke and 2020B in Kole.

The number of *S. frugiperda* moths trapped per field differed significantly between districts (F_2,123_ = 28.86, *p* < 0.001) and season within districts (F_6,123_ = 2.59, *p* = 0.02), and not with growth stages (F_3,123_ = 2.22, *p* = 0.09). The highest numbers of adults were trapped in Kiryandongo (7.4 ± 1.29 moths per field), followed by Nakaseke (3.5 ± 0.80 moths per field), while the lowest numbers were trapped in Kole (1.4 ± 0.39 moths per field). When compared between growth stages in the different districts in the different seasons, significant differences only occurred in Kole in 2021A (F_3,17_ = 5.37, *p* = 0.016), where more adults were recorded at the late vegetative stage (Table 3).

Maize in all investigated fields was damaged by *S. frugiperda*. Leaf damage varied significantly between the districts (F_2,167_ = 16.6, *p* < 0.001) and seasons within a district (F_6,167_ = 3.77, *p* = 0.002). Leaf damage was highest in Kiryandongo (2.4 ± 0.14), followed by Kole (2.0 ± 0.11), and lowest in Nakaseke (1.8 ± 0.10). Leaf damage was highest in 2020B and 2021B in Kiryandongo and in 2021A and 2021B in Kole and Nakaseke (Table 3). Damage incidence was significantly higher in Nakaseke (87.7 ± 1.19) than in Kiryandongo (81.8 ± 2.55) and Kole (79.1 ± 2.95). Damage incidence only varied significantly between seasons in Nakaseke, where the lowest incidence occurred in 2020B (Table 4).

### 3.2. Variation in Spodoptera frugiperda Abundance and Damage with Maize Growth Stage

There were significant differences between maize growth stages in the number of egg masses (F_3,167_ = 4.70, *p* = 0.003), larval abundance (F_3,167_ = 43.16, *p* < 0.001), leaf damage (F_3,167_ = 89.97, *p* < 0.001) and damage incidence (F_3,167_ = 65.57, *p* < 0.001).

The mean number of egg masses was significantly higher at the late vegetative stage (0.03 ± 0.12 egg masses per 15 plants) but was similar at the early vegetative (0.02 ± 0.01 egg masses per 15 plants), tasselling (0.01 ± 0.01 egg masses per 15 plants) and reproductive stages (0.01 ± 0.00 egg masses per 15 plants).

**Figure 2:**
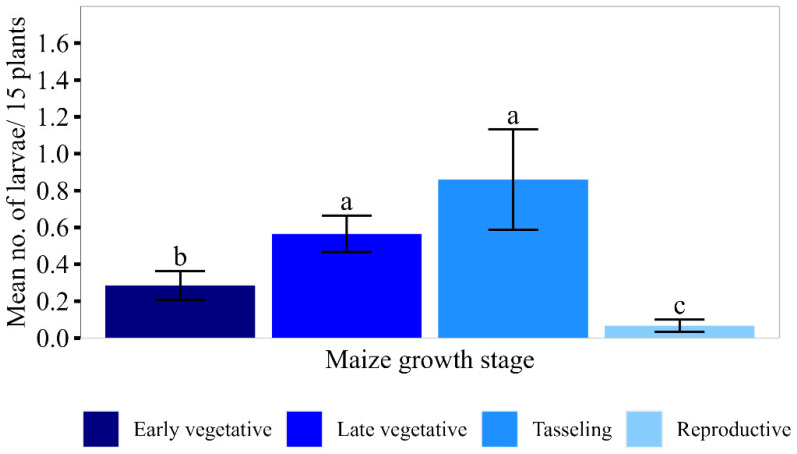
The mean number (±SEM) of *Spodoptera frugiperda* larvae per 15 plants at different maize growth stages (pooled for seasons and districts). Means having similar letters are not significantly different at *p* < 0.05.

**Figure 3:**
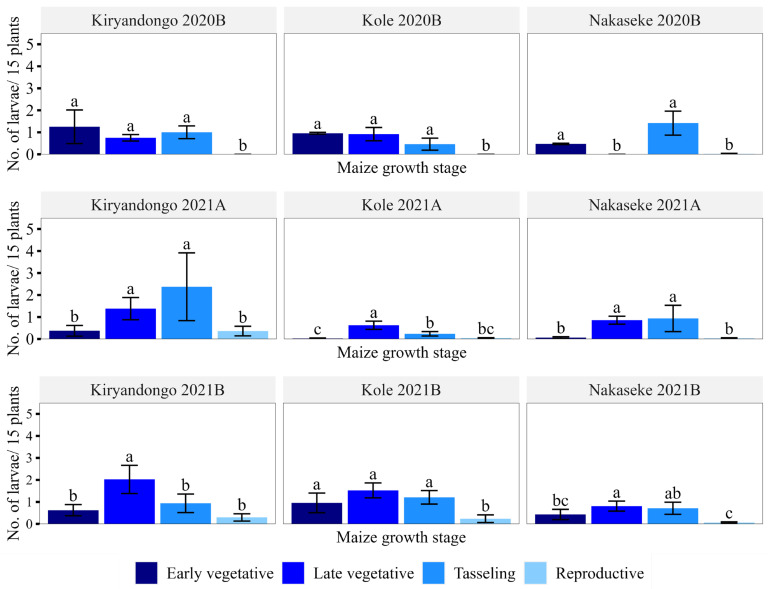
Mean number (±SEM) of *Spodoptera frugiperda* larvae per 15 plants at different maize growth stages in the surveyed seasons and districts. Means are separated for growth stages per district in a given season. Means having similar letters are not significantly different at *p* < 0.05. Each year’s first and second rainy seasons are distinguished by the letters A and B, respectively.

Overall larval abundance was lowest at the reproductive stage, followed the early vegetative stage, whilst the late vegetative and tasselling stages had similar larval numbers (Figure 2). When separated by district, the lowest larval abundance occurred at the tasselling stage in all seasons and at the early vegetative stage in two seasons in Kiryandongo and Nakaseke (Figure 3). The abundance of larvae in the late vegetative and tasselling stages was comparable in most cases; the only exceptions occurred in Kiryandongo in 2021B and Kole in 2021A, when significantly higher larval abundance occurred at the late vegetative stage than at tasselling (Figure 3). In Nakaseke, however, more larvae were recorded at tasselling than at the late vegetative stage.

**Figure 4:**
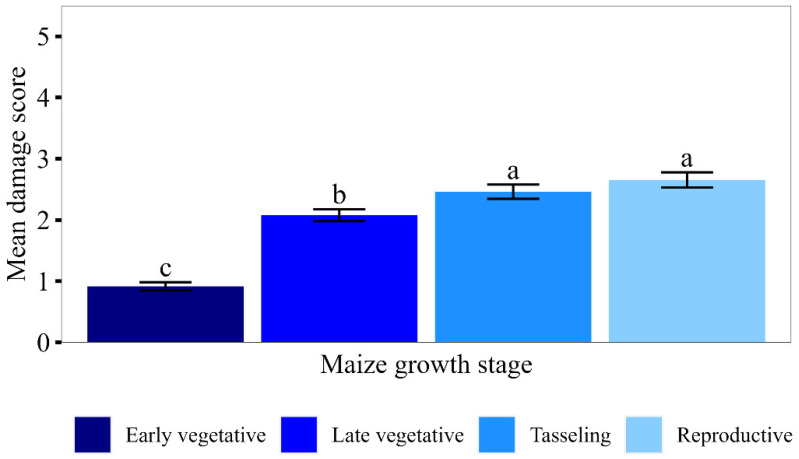
Mean leaf damage (±SEM) at different growth stages (pooled over seasons and districts). Means having similar letters are not significantly different at *p* < 0.05.

**Figure 5:**
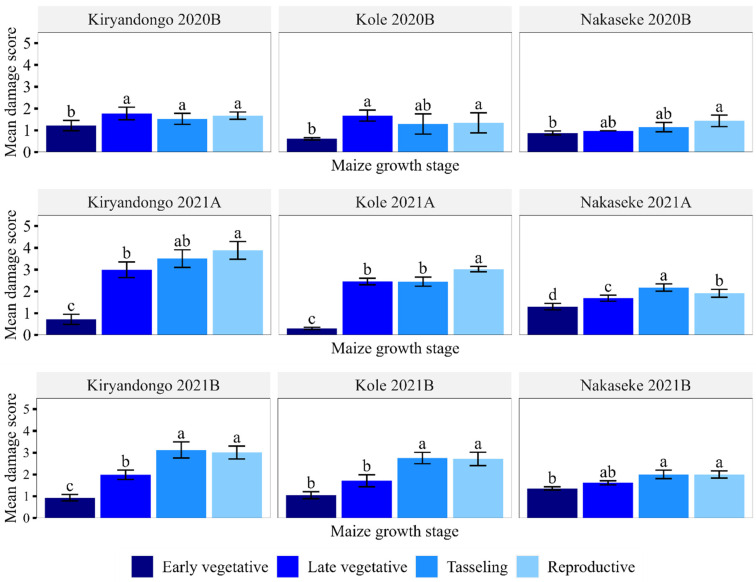
Mean leaf damage (±SEM) at different growth stages in the surveyed seasons and districts. Means are separated for growth stage per district in a given season. Means having similar letters are not significantly different at *p* < 0.05. Each year’s first and second rainy seasons are distinguished by the letters A and B, respectively.

Overall, leaf damage increased with the maize crop age and was significantly lower at the early vegetative stage, followed by the late vegetative stage. Damage at tasselling and reproductive stages was higher and similar (Figure 4). The damage pattern was similar in all seasons in all the districts (Figure 5). Damage incidence increased with crop age and was significantly lower at the early vegetative stage (54.6 ± 3.25), followed by the late vegetative stage (86.4 ± 1.93). The highest damage incidence occurred at the tasselling (93.4 ± 1.13) and reproductive stages (94.5 ± 1.05).

### 3.3. The Relationship between Management Practices and Leaf Damage/Larval Abundance

We conducted regression analyses on the relationship between farm management practices and weather factors on the abundance of *S. frugiperda* larvae and damage (Supplementary Tables S1 and S2).

Pesticide use frequency, weeding frequency, cropping system, and maximum temperature were not significant predictors for the abundance of *S. frugiperda* larvae. However, fertilizer use, tillage system and rainfall were significant predictors of larval abundance. Shifts from no fertilizer to fertilizer use in Nakaseke in 2021A and conservation tillage to conventional tillage in Nakaseke in 2021B, and high rainfall in both Kole and Nakaseke in 2021B, increased larval abundance (Supplementary Tables S1 and S2).

Pesticide use frequency and cropping system were insignificant leaf damage and larval abundance predictors. Fertilizer use, rainfall and maximum temperature were significant predictors leading to a high larval abundance. Fertilizer use, tillage system, weeding frequency, rainfall and maximum temperature were significant predictors of leaf damage (Supplementary Tables S1 and S2). A shift from no fertilizer use to fertilizer increased leaf damage in Nakaseke (2021A) and Kole (2021B), while the converse was observed in Kiryandongo in 2021B. Increased weeding frequency was associated with high *S*. *frugiperda* leaf damage in Kiryandongo (2020B) and Nakaseke (2021A) (Supplementary Tables S1 and S2). Shifting from conservation tillage to conventional tillage increased leaf damage in Nakaseke (2020B) and Kiryandongo (2021B). Rainfall was a significant predictor for *S. frugiperda* leaf damage in Kiryandongo in all seasons, the two seasons of 2021 in Kole, and 2021B in Nakaseke. Maximum temperature significantly predicted *S. frugiperda* leaf damage in Kiryandongo and Kole in season 2021A and Nakaseke 2021B only where an increase in maximum temperature decreased leaf damage.

## 4. Discussion

This study aimed to assess population dynamics and damage by *S. frugiperda* as influenced by maize phenology, farmers’ practices and environmental conditions in three districts of Uganda. The larval numbers depended on district, season, fertilizer use, tillage system, and rainfall, while the number of eggs depended on district and season. *Spodoptera frugiperda* leaf damage depended on district, season, tillage system, fertilizer use, weeding frequency, rainfall, and maximum temperature. The differences in districts and seasons are attributed to differences in weather factors (rainfall and temperature) and the main agronomic practices.

### 4.1. Spodoptera frugiperda Abundance and Damage as Influenced by Maize Growth Stage

The results of this study have shown that the number of larvae was generally higher at the late vegetative and tasselling stages. A reasonably similar pattern was observed in the populations of adult moths. An increase in larval numbers in late vegetative and tasselling stages may be due to the immigration of adult moths from neighbouring fields and an increase in the populations of individuals in the fields. These observations are similar to those reported by Niassy et al. [20], who reported high infestation of maize at the vegetative and reproductive stages.

Leaf damage generally peaked in the late vegetative stage, tasselling or reproductive stages. The increase in leaf damage with the growth stage may be because of the gradual build-up in the larval population and the increase in the abundance of older larvae that eat proportionate to their weight [21]. Also, increased damage with crop age may result from an influx of new moths and multiplication in the same fields. It was also reported in Egypt that *S. frugiperda* damage increased with an increase in maize age [21]. Gross et al. [22] mentioned that the sensitivity of maize growth stages to *S. frugiperda* attack varied based on the plant growth and development. These results imply that farmers must regularly and closely monitor maize fields to intervene and prevent the population of *S. frugiperda* from reaching economically damaging levels at the vegetative or reproductive stages.

### 4.2. The Abundance and Damage by Spodoptera frugiperda as Influenced by Management Practices and Weather Factors

Pesticides are the most popular management option for *S. frugiperda*. In our study, however, we did not realize any differences in leaf damage under the different pesticide spray frequencies. Pesticide application also did not cause a significant reduction in larval numbers. This may be because of the timing of application, whereby fields could have been sprayed late when the damage was already high, or because the application rate of the pesticides was below the recommended rate, as was reported by most farmers in Kiryandongo. For instance, an average of 8 mls/20 L of Roket (Profenofos and Cypermethrin) was used, below the recommended rate of 30 mls/20 L. Kalyebi et al. [11] reported inconsistent efficacy of pesticides used by farmers and attributed it to differences in the types, doses, and frequency of pesticide application. Kansiime et al. [23] reported that pesticide application often led to increased yield. This is consistent with our results in Uganda [24], where application of pesticides decreased leaf damage and resulted in higher yields. More marked reductions were, however, observed when application started at 10 to 20 days after emergence [8].

This study showed that fertilizer use significantly predicted *S. frugiperda* leaf damage, where fertilizer use sometimes increased or reduced damage. The inconsistencies in the influence of fertilizers may be due to the type and quantity of fertilizer used. For instance, Fiaboe et al. [25] recently reported that split application of NPK reduced *S. frugiperda* incidence and damage. Nitrogenous fertilizers change the C/N ratio and make plants more susceptible to *S. frugiperda* damage [26]. A reduction in damage in fields where fertilizers are applied may be because fertilizers enhance plant vigour and make them more resilient to pest attacks. More controlled studies will be required to assess the effect of different fertilizers on *S. frugiperda* incidence and damage.

Leaf damage was significantly different among the different weeding frequencies. An increase in weeding frequency increased damage in Kiryandongo and Nakaseke. During data collection, we noticed that fields with many weeds had less damage because *S. frugiperda* moths prefer to lay their eggs in well-weeded, healthy green maize. Poor or unweeded maize tends to be yellowish and less preferred by ovipositing moths. In contrast to our findings, studies have shown that repeated weeding reduced *S. frugiperda* damage, probably due to a reduction in the abundance of host plants [27]. These inconsistencies could also be because of the differences in the diversity of weed species, weeding operations and interaction of other management practices in farmers’ fields. This calls for more controlled experiments to assess the influence of weeds on *S. frugiperda* infestation and damage.

Fields with conservation tillage had lower *S. frugiperda* damage and larval abundance than those using conventional tillage practices. This was also observed in Zimbabwe, where minimum or no tillage significantly reduced *S. frugiperda* infestation [27]. Maize production under zero or minimum tillage was reported to reduce *S. frugiperda* damage in the Americas because it favoured population build-up of predatory species. Most farmers in Nakaseke practice conservation tillage, and their fields had the highest larval parasitism rates (3.3%) [28]. This partly explains the low abundance of larvae in this district.

Intercropping did not influence *S. frugiperda* larval abundance and leaf damage. This contradicts research carried out by Yigezu and Wakgari [29], where intercropping with non-host legumes such as beans significantly reduced *S. frugiperda* infestation. Also in support of the findings of this study, intercropping with legumes, such as cowpea (*Vigna unguiculata* L.), groundnut (*Arachis hypogaea* L.), and common bean (*Phaseolus vulgaris* L.), was ineffective in reducing *S. frugiperda* damage [30]. In the current study, the intercrops included beans, soybean, peas, groundnuts, simsim, and cassava, with most of the fields being intercropped with soybeans.

In several cases, there were significant negative relationships between rainfall and *S. frugiperda* damage. Heavy rains increase egg and larval dislodgement and trapping and drowning of moths in pupation tunnels and reduce pupal survival and emergence [31,32]. The negative impact of rainfall could mean that farmers who plant early can exploit the nitrogen flush for high vigour and avoid severe damage by *S. frugiperda*. Exploiting the period of much rainfall also results in a reduction in pesticide use and, therefore, a reduction in the cost of production and harmful effects associated with pesticide use.

Maximum temperature had a significant negative influence on *S. frugiperda* leaf damage. However, maximum temperature had no significant relationship with the abundance of *S. frugiperda* larvae. While high temperature rises are reported to favour *S. frugiperda* multiplication and potentially lead to higher damage levels, the increase is within limits. The temperatures recorded during our study were between 26 °C and 36 °C, and as reported by Savadatti et al. [33], temperatures beyond 32 °C result in immediate mortality of enclosed adults.

## 5. Conclusions and Recommendations

The study showed significant differences between districts and growth stages in *S. frugiperda* abundance and damage. Kiryandongo had the highest leaf damage and Nakaseke the lowest. The late vegetative to ripening stages had the highest abundance of *S. frugiperda* larvae, while the late vegetative to tasselling stages had the highest mean leaf damage. Fertilizer use, tillage system and rainfall were the only significant predictors for *S. frugiperda* larval abundance. A shift from conservation to conventional tillage, no fertilizer to fertilizer use, and low to high rainfall increased the abundance of *S. frugiperda* larvae. There was one case where high rainfall led to increased larval abundance. The mean number of larvae was not significantly associated with the mean daily maximum temperature in any district. Reduced weeding frequency, conservation tillage, high rainfall and high maximum temperature were associated with reduced *S. frugiperda* leaf damage. The influence of fertilizer use on leaf damage was contradictory, leading to reduced or increased leaf damage. Pesticide application frequencies, and cropping systems, were not associated with *S. frugiperda* damage.

Monitoring and scouting of maize fields should start immediately after maize crop emergence, since *S. frugiperda* infestation was recorded from the early vegetative to reproductive stages. Sensitization of farmers to be more vigilant in monitoring and scouting for *S. frugiperda* when there is less or no rain would be helpful. In addition, exploiting the use of natural controls through the integration of weather information in *S. frugiperda* management could help reduce unnecessary pesticide applications, save costs for farmers, and reduce heavy environmental hazards. There is a need to promote conservation tillage to reduce *S. frugiperda* abundance in maize fields. Our study involved monitoring of farmers’ fields under no control of the studied factors. While this provided good information on the abundance and severity of damage by *S. frugiperda,* inconsistencies were observed in the influence of different factors. This may be because of the heterogeneity in farmer practices and the environment. Therefore, there is a need to conduct more controlled studies to evaluate the effect of management practices (pesticide use, fertilizer use, cropping system, and weeding frequency) on the abundance of and damage to *S. frugiperda* and to determine the yield loss relationship in order to develop economic injury levels and thresholds for fall armyworm in Uganda.

## Figures and Tables

**Table 1:** Characteristics of the different districts where the population dynamics of *Spodoptera frugiperda* studies were conducted.

District	Mean Annual Rainfall (mm)	Altitude (Meters Masl)	Mean Daily Temp. (°C)	Soil Type	Crop Growing Months	Major Crops Grown
Kole	1283	1072	Max: 31.6 °C Min: 19.5 °C	Sandy clay loam	April–October	Maize Millet Cassava Beans
Nakaseke	1728	1200	Max: 29.5 °C Min: 18.5 °C	Sandy clay loam	March–May August–November	Bananas Coffee Potatoes Beans
Kiryandongo	1153	1160	Max: 31.8 °C Min: 19.8 °C	Sandy loam	March–May August–November	Maize Cassava Beans Sweet potatoes

Source: https://weatherspark.com/countries/UG, accessed on 23 September 2021, https://www.nakaseke.go.ug/about-us/district-profile, accessed on 30 September 2021, https://www.kole.go.ug/about-us/district-profile, accessed on 30 September 2021, https://www.kiryandongo.go.ug/about-us/district-profile accessed on 30 September 2021.

**Table 2:** Abundance (mean ± SEM) of *Spodoptera frugiperda* life stages by district and season.

District/Season	Mean No. of Egg Masses Per 15 Plants			Mean No. of Larvae Per 15 Plants		
	Kiryandongo	Kole	Nakaseke	Kiryandongo	Kole	Nakaseke
2020B	0.02 ± 0.019 a	0.10 ± 0.066 a	0.00 ± 0.000 b	1.00 ± 0.302 a	0.78 ± 0.195 a	0.76 ± 0.293 a
2021A	0.01 ± 0.007 a	0.02 ± 0.008 a	0.05 ± 0.016 a	1.10 ± 0.412 a	0.24 ± 0.065 b	0.57 ± 0.181 a
2021B	0.00± 0.001 a	0.02 ± 0.009 a	0.00 ± 0.000 b	0.07 ± 0.015 b	0.06 ± 0.011 b	0.03 ± 0.007 b
Overall mean	0.01	0.03	0.02	0.68	0.25	0.36
*p*-value	0.226	0.17	0.019	<0.001	<0.001	<0.001
F statistics	1.53	1.832	4.31	11.697	9.608	10.576
Df (season, residual)	2,55	2,57	2,49	2,55	2,57	2,49
CV (%)	250.1	184.7	268.2	55.7	68.7	68.7

For each variable, means within a column followed by similar letters are not significantly different at *p* < 0.05. Each year’s first and second rainy seasons are distinguished by the letters A and B, respectively.

**Table 3:** Mean number (±SEM) of *Spodoptera frugiperda* moths trapped per field by growth stage, season, and district.

Maize Growth Stage	Kiryandongo				Kole				Nakaseke				Grand Mean
	2020B	2021A	2021B	Mean	2020B	2021A	2021B	Mean	2020B	2021A	2021B	Mean	
Early vegetative	11.5	2.0	-	6.8	1.0	-	-	1.0	0.4	1.0	2.5	1.3	3.1
Late vegetative	7.0	9.2	5.5	7.2	0.0	1.2 a	8.4	3.2	0.0	1.6	7.6	3.1	3.6
Tasselling	2.7	7.7	5.3	5.2	0.3	0.3 b	4.9	1.8	0.0	2.9	6.1	3.0	4.3
Reproductive	3.0	9.6	6.5	6.4	1.3	0.6 b	2.1	1.3	0.1	9.1	2.4	3.9	2.5
Grand mean	6.1	7.1	5.8	6.4	0.7	0.7	5.1	1.8	0.1	3.7	4.7	2.8	3.4
Se	2.1	1.8	0.3	1.8	0.3	0.2	0.9	0.8	0.2	1.9	0.8	1.5	1.6
*p*-value	0.149	0.102	0.600	0.634	0.281	0.016	0.057	0.634	0.075	0.975	0.297	0.635	0.307
F statistics	2.57	2.41	0.54	0.61	1.62	5.37	3.54	0.06	5.09	0.03	1.36	0.61	1.21
Df (growth stage, residual)	3,6	3,17	3,10	3,38	3.6	3,17	3,14	3,41	3,4	3,16	3,13	3,38	3,162

For each variable, means within a column followed by similar letters are not significantly different at *p* < 0.05. Each year’s first and second rainy seasons are distinguished by the letters A and B, respectively.

**Table 4:** Leaf damage severity (±SEM) and damage incidence (±SEM) by district and season.

District/ Season	FAW Mean Leaf Damage Score			Damage Incidence (%)		
	Kiryandongo	Kole	Nakaseke	Kiryandongo	Kole	Nakaseke
2020B	2.1 ± 0.160 b	1.6 ± 0.253 b	1.5 ± 0.142 b	85.7 ± 3.13 a	73.9 ± 6.12 a	78.2 ± 2.77 b
2021A	2.7 ± 0.264 a	2.0 ± 0.181 a	1.8 ± 0.089 a	83.5 ± 4.72 a	79.8 ± 5.55 a	91.4 ± 1.490 a
2021B	2.0 ± 0.178 b	2.1 ± 0.167 a	1.8 ± 0.080 a	78.4 ± 3.32 a	80 ± 3.31 a	86.9 ± 1.93 a
Overall mean	2.4	2	1.8	81.8	79.1	87.7
*p*-value	<0.001	0.077	0.02	0.193	0.524	0.005
F statistics	9.168	2.69	4.24	1.697	0.654	5.943
Df (season, residual)	2,55	2,57	2,49	2,55	2,57	2,49
CV (%)	29.7	29.7	17.9	17.1	21.5	12.3

For each variable, means within a column followed by similar letters are not significantly different at *p* < 0.05. Each year’s first and second rainy seasons are distinguished by the letters A and B, respectively.

## Data Availability

The data presented in this study are available on request from the corresponding author.

## Supplementary Materials

The following are available online at https://www.mdpi.com/article/10.3390/insects15050301/s1. Table S1: Multiple linear regression analysis coefficients of the relationship between farm management practices and weather factors with damage and abundance of *Spodoptera frugiperda* larvae in 2020B and 2021A. Table S2: Multiple linear regression analysis coefficients of the relationship between farm management practices and weather factors with damage and abundance of *Spodoptera frugiperda* larvae in 2021B.
