# Supplementary material for: Population Dynamics of Fall Armyworm (Lepidoptera: Noctuidae) in Maize Fields in Uganda
Source: Insects. 2024 Apr 23;15(5):301. doi: 10.3390/insects15050301 (PMC11122130; doi:10.3390/insects15050301)
Supplement: Supplementary file 1 [file insects-15-00301-s001.zip › insects-2824968-supplementary.pdf]

Supplementary Table S1: Multiple linear regression analysis coefficients of the relationship between farm management practices and weather factors with damage and abundance of *Spodoptera frugiperda* larvae in 2020B and 2021A

Location	Variable	Mean number of larvae per 15 plants				Mean leaf damage			
		Estimate	Std. Error	t value	Pr(>|t|)	Estimate	Std. Error	t value	Pr(>|t|)
**2020B**									
**Kiryandongo**	(Intercept)	0.18	0.224	0.788	0.440	1.30	2.054	0.632	0.535
	Pesticide use frequency	-	-	-	-	-	-	-	-
	Fertilizer use	-	-	-	-	-	-	-	-
	Weeding frequency	0.02	0.030	0.683	0.502	0.63	0.279	2.260	0.035*
	Tillage system	-	-	-	-	-	-	-	-
	Cropping system	-	-	-	-	-	-	-	-
	Rainfall	0.00	0.001	-0.644	0.527	-0.02	0.005	-3.395	**0.003****
	Maximum temperature	-0.01	0.007	-0.830	0.417	0.02	0.060	0.366	0.718
**Kole**	(Intercept)	0.40	0.531	0.750	0.462	-2.53	4.314	-0.586	0.564
	Pesticide use frequency	-	-	-	-	-	-	-	-
	Fertilizer use	-	-	-	-	-	-	-	-
	Weeding frequency	-	-	-	-	-	-	-	-
	Tillage system	-	-	-	-	-	-	-	-
	Rainfall	0.00	0.001	-0.093	0.927	0.00	0.007	-0.467	0.645
	Cropping system	-	-	-	-	-	-	-	-
	Maximum temperature	-0.01	0.015	-0.713	0.484	0.13	0.119	1.081	0.292
**Nakaseke**	(Intercept)	0.00	0.000	- -	- -	2.02	2.709	0.747	0.466
	Pesticide use frequency	0.00	0.000	- -	- -	0.06	0.150	0.380	0.709
	Fertilizer use	-	-	-	-	-	-	-	-
	Weeding frequency	-	-	-	-	-	-	-	-
	Tillage system	0.00	0.000	- -	- -	0.70	0.301	2.329	0.033 *
	Cropping system	-	-	-	-	-	-	-	-
	Rainfall	0.00	0.000	- -	- -	-0.01	0.006	-0.870	0.397
	Maximum temperature	0.00	0.000	- -	- -	-0.01	0.076	-0.184	0.857
**2021A**									
**Kiryandongo**	(Intercept)	1.47	2.044	0.720	0.476	73.09	19.946	3.664	0.0001***
	Pesticide use frequency	0.00	0.039	-0.076	0.940	-0.27	0.385	-0.702	0.487
	Fertilizer use	-0.03	0.062	-0.467	0.643	0.69	0.608	1.126	0.267
	Weeding frequency	0.05	0.062	0.844	0.403	0.12	0.608	0.189	0.851
	Tillage system	0.08	0.063	1.209	0.233	0.88	0.611	1.435	0.159
	Cropping system	-0.08	0.048	-1.672	0.102	-0.63	0.472	-1.343	0.186
	Rainfall	0.00	0.002	-0.806	0.425	-0.08	0.016	-4.953	0.1 e^{-4}***
	Maximum temperature	-0.05	0.062	-0.761	0.451	-2.12	0.606	-3.497	0.001**
**Kole**	(Intercept)	-0.37	0.363	-1.028	0.310	38.40	15.071	2.548	0.014*
	Pesticide use frequency	0.01	0.011	0.854	0.398	0.25	0.449	0.552	0.584
	Fertilizer use	-0.01	0.011	-1.130	0.265	-0.21	0.475	-0.447	0.657
	Weeding frequency	0.00	0.017	-0.225	0.823	-0.07	0.712	-0.096	0.924
	Tillage system	-	-	-	-	-	-	-	-
	Cropping system	-0.02	0.011	-1.804	0.078	-0.43	0.475	-0.902	0.372
	Rainfall	0.00	0.000	1.809	0.077	-0.03	0.007	-3.587	0.1 e^{-3}***
	Maximum temperature	0.01	0.012	1.088	0.283	-1.12	0.479	-2.342	0.024*
**Nakaseke**	(Intercept)	0.33	0.189	1.724	0.092	0.86	1.675	0.513	0.610
	Pesticide use frequency	-0.01	0.019	-0.318	0.752	-0.23	0.166	-1.364	0.180
	Fertilizer use	0.10	0.044	2.193	0.034*	1.34	0.394	3.410	0.001**
	Weeding frequency	0.01	0.011	1.271	0.211	0.20	0.100	2.026	0.049*
	Tillage system	0.00	0.016	0.035	0.972	-0.06	0.145	-0.398	0.692
	Cropping system	-	-	-	-	-	-	-	-
	Rainfall	0.00	0.000	-0.985	0.330	0.00	0.002	-1.125	0.267
	Maximum temperature	-0.01	0.006	-1.717	0.093	0.04	0.055	0.663	0.511

Note: (-) Note applicable because only one alternative of a given management practice was done in a given location and season. (- -) No larvae were recovered. Each year's first and second rainy seasons are distinguished by the letters A and B, respectively. *, ** and *** denote significance at 0.5, 0.1 and 0.01%, respectively.

Supplementary Table S2: Multiple linear regression analysis coefficients of the relationship between farm management practices and weather factors with damage and abundance of *Spodoptera frugiperda* larvae in 2021B

Location	Variable	Mean number of larvae per 15 plants				Mean leaf damage			
		**Estimate**	**Std. Error**	**t value**	**Pr(>|t|)**	**Estimate**	**Std. Error**	**t value**	**Pr(>|t|)**
**2021B**									
Kiryandongo	(Intercept)	0.29	0.382	0.750	0.457	4.96	2.029	2.444	0.018*
	Pesticide use frequency	-0.02	0.071	-0.294	0.770	0.43	0.376	1.153	0.254
	Fertilizer use	-0.06	0.071	-0.883	0.381	-0.82	0.376	-2.173	0.034*
	Weeding frequency	-0.01	0.050	-0.208	0.836	0.38	0.266	1.411	0.164
	Tillage system	0.06	0.058	1.082	0.284	1.15	0.307	3.747	$0.4e^{-3}$***
	Cropping system	-	-	-	-	-	-	-	-
	Rainfall	0.00	0.001	1.791	0.0789	-0.03	0.004	-6.996	$0.4e^{-8}$***
	Maximum temperature	-0.01	0.012	-0.786	0.436	-0.11	0.064	-1.751	0.086
Kole	(Intercept)	0.12	0.546	0.228	0.821	4.11	2.704	1.521	0.136
	Pesticide use frequency	-0.02	0.051	-0.298	0.767	-0.40	0.253	-1.583	0.121
	Fertilizer use	0.00	0.050	-0.044	0.965	0.83	0.246	3.389	0.002**
	Weeding frequency	0.03	0.045	0.721	0.475	0.41	0.224	1.815	0.077
	Tillage system	-	-	-	-	-	-	-	-
	Cropping system	-0.03	0.060	-0.434	0.666	-0.31	0.297	-1.053	0.298
	Rainfall	0.00	0.001	2.398	**0.021 ***	-0.03	0.006	-4.643	$0.323e^{-4}$***
	Maximum temperature	-0.01	0.018	-0.311	0.757	-0.04	0.088	-0.485	0.630
Nakaseke	(Intercept)	-0.17	0.212	-0.820	0.416	5.63	1.122	5.016	$0.6e^{-5}$***
	Pesticide use frequency	0.00	0.026	0.147	0.884	-0.16	0.137	-1.139	0.260
	Fertilizer use	-0.04	0.036	-1.166	0.249	0.14	0.189	0.757	0.452
	Weeding frequency	-0.05	0.033	-1.503	0.139	0.06	0.173	0.361	0.719
	Tillage system	0.07	0.033	2.054	**0.045***	0.39	0.176	2.192	0.033*
	Rainfall	0.00	0.001	-0.439	0.663	-0.01	0.003	-3.824	$0.3e^{-3}$***
	Cropping system	-0.09	0.052	-1.760	0.084	-0.10	0.273	-0.351	0.727
	Maximum temperature	0.01	0.007	1.380	0.174	-0.12	0.038	-3.105	0.003**

Note: (-) Note applicable because only one alternative of a given management practice was done in a given location and season. Each year's first and second rainy seasons are distinguished by the letters A and B, respectively. *, ** and *** denote significance at 0.5, 0.1 and 0.01%, respectively.
